# Effect of low-level laser therapy on osseointegration of titanium dental implants in ovariectomized rabbits: biomechanics and micro-CT analysis

**DOI:** 10.1186/s40729-020-00257-z

**Published:** 2020-10-12

**Authors:** Mustafa Karakaya, Ahmet Emin Demirbaş

**Affiliations:** 1 Sancaktepe Oral and Dental Health Hospital, Department of Oral and Maxillofacial Surgery, Ministry of Health, İstanbul, Turkey; 2 Department of Oral and Maxillofacial Surgery, Erciyes University Faculty of Dentistry, Melikgazi, Kayseri, Turkey

**Keywords:** Dental implant, Osseointegration, Low-level laser therapy, Osteoporosis, Micro-CT

## Abstract

**Purpose:**

The primary aim of this study is to assess, in an animal model, whether biostimulation of osteoporotic bone with low-level laser therapy improves the osseointegration of dental implants.

**Material and methods:**

Twenty-two female rabbits were randomly divided into two groups: sham-ovariectomy and bilateral-ovariectomy. Laser therapy was applied to the implants placed in the right tibial bones and was not applied to implants placed in the left tibial bones. The periotest device was used for the stability test. Periotest values were recorded after the implantation (T0) and when the animals were euthanized (T1). The removal torque test and micro-computed tomography examination were evaluated.

**Results:**

As a result of removal torque, the mean of ovariectomy-laser group (56.1 ± 5.1 Ncm) was higher than sham-ovariectomy group (55.4 ± 18.5 Ncm) (*p* = 0.9). In periotest analysis, a significant difference was found between the values of T1 and T0 in all groups, except sham-ovariectomy group (*p* < 0.05); and the highest difference was found in the ovariectomy-laser group. Micro-CT examination demonstrated that ovariectomy-laser group showed an increase of implant–bone contact when compared with ovariectomy (*p* < 0.05).

**Conclusions:**

The values obtained from biomechanical tests and micro-CT in the ovariectomy-laser group were significantly higher than the ovariectomy group and achieved the values in the healthy bone.

## Introduction

Dental implants are frequently used in prosthetic treatment with the aim of restoring the loss of esthetic and function. Osseointegration is very important in the success of dental implants and is defined as “the direct structural and functional connection between a load-bearing implant surface and bone” [1]. One of the factors influencing the osseointegration is the quality of the bone surrounding the implant. In the presence of intense bone, the percentage of implant-to-bone contact increases and the implant is more stable during recovery [2]. Low or poor bone quality and insufficient primary stabilization are important reasons for implant failure. Osteoporosis is one of the diseases that cause low bone quality. It has been defined as a systemic skeletal disease characterized by low bone mass and microarchitectural deterioration of bone tissue, with consequent increase in bone fragility and susceptibility to fracture [3]. Osteoporosis-related changes in the jaws are not different from the changes in the other bones of the body [4]. Bone density and quality in aging and osteoporosis are negatively affected by the decrease in cell proliferation, cellular synthesis activity, cellular sensitivity, and in the number of mesenchymal stem cells [5]. Decreased bone mass and bone mineral density have been reported to cause delayed healing of fractures and bone repair [6]. This increases the risk of failure in the integration of any biomaterial implanted into the osteoporotic bone. The need for developing different treatment modalities for the treatment of osteoporotic fractures, improving the osseointegrations of biomaterials applied to those bones, and reducing the risks of failed osseointegrations is critical. Low-level laser therapy (LLLT) as a promising treatment option induces osteogenesis and contributes positively to bone healing [7]. LLLT has been successfully used to improve bone healing after tooth extraction and fractures of bones to accelerate orthodontic tooth movement [8, 9]. The stimulating effect of LLLT on bone is related to proliferation of fibroblasts and osteoblasts during mesenchymal differentiation. LLLT has also been reported to increase the number of collagen fibers in bones [10]. The increase in tissue vascularization by LLLT stimulates the production of bone matrix and improves bone healing by the release of mediators [8, 10, 11]. These positive effects of LLLT on bone healing are thought to improve osseointegration of dental implants in low-density bones.

The primary aim of this study is to increase the osseointegration of dental implants applied to the osteoporotic bone with the biostimulatory effect of LLLT.

## Materials and methods

### Animals

Twenty-two New Zealand (*Oryctolagus cuniculus L.*) adult female rabbits aged 8 months were used in the study. At the beginning of the study, veterinarians performed a health check. All animal procedures were approved by the Erciyes University Experimental Animal Ethics Committee and performed in compliance with the guidelines for the care and handling of experimental animals of the medical research center at Erciyes University, Kayseri, Turkey.

### Group design

**Fig. 1:**
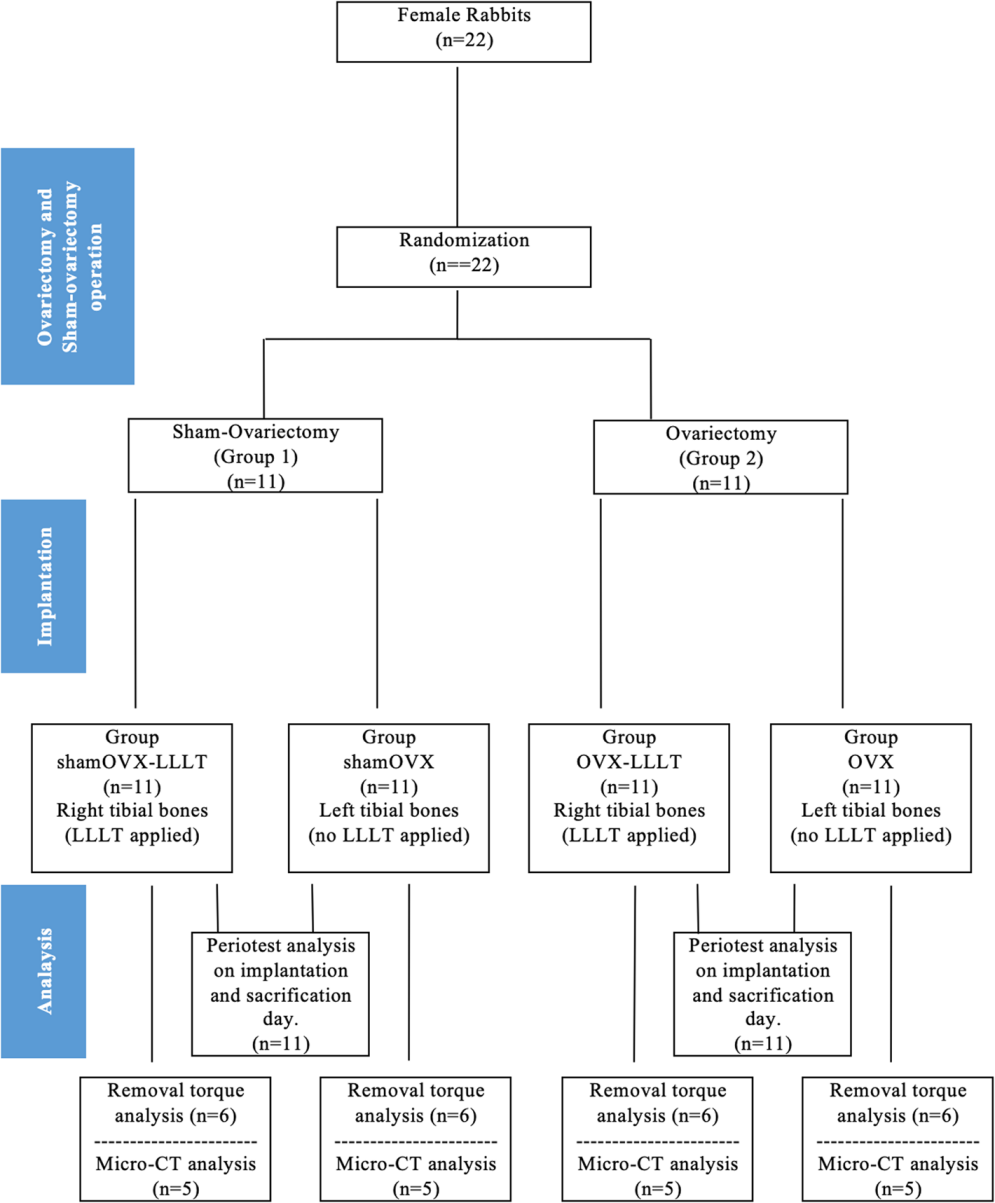
Group design

The rabbits were randomly divided into two groups: sham ovariectomy (shamOVX, *n*:11) and bilateral ovariectomy (OVX, *n*:11) (Fig. 1). LLLT was applied to the right tibial bones of each rabbit, but not to the left tibial bones. Group shamOVX was divided into two subgroups after ovariectomy and implantation: group shamOVX-LLLT (*n*:11) and group shamOVX (*n*:11). And group OVX was divided into two subgroups after ovariectomy and implantation: group OVX-LLLT (*n*:11) and group OVX (*n*:11).

### Experimental design

A total of 44 (4.1 × 6 mm) tissue-level SLA implant (Bilimplant®, Turkey) placements were planned at the left and right tibia metaphysis of all rabbits. In the second (OVX) group, ovariectomy was performed before implantation, and an osteoporosis model was established to reduce bone quality and density. Ovariectomy was performed under general anesthesia with intramuscular ketamine (Ketalar® 50 mL, 50 mg/mL, Pfizer, UK) and xylazine (Rompun® 25 mL, 100 mg/mL, Bayer HealthCare, USA). After 2 weeks, methylprednisolone (Prednol-1® 40 mg, 1 ampoule, Mustafa Nevzat, Turkey) was given intramuscularly for 4 weeks with 1 mg/kg/day to accelerate osteoporosis formation. At the end of this 6-week period, the implants were placed in the second group. To prevent the development of infection, 50 mg/kg of cefazolin (Cefamezin® IM Vial, Eczacibasi, Turkey) was injected intramuscularly in the pre- and postoperative periods for 3 days. Also, 1 mg/kg diclofenac (Diklofen®, Turkey) as an analgesic was injected intramuscularly for pain control. Both groups were subdivided into two subgroups, and LLLT was applied to the implants placed in the right tibial bone, whereas the left tibial bone was not applied (Fig. 1). A 940-nm wavelength diode laser (BIOLASE Technology Inc., Irvine, CA, USA) was used for LLLT. The laser device was used as a continuous wave at a 0.3 W output power and was applied at a distance of 1.5 cm from the implants. Implants were exposed to LLLT from four points at 20 s/cm^2^ with at least 6 J/cm^2^ of energy and 0.3 W of power. Laser application was started immediately after surgery and was continued for 10 days. Six weeks after the implants placement, rabbits were euthanized by induction of respiratory depression with high doses of anesthetic agents. The tibial bones in which the implants were placed were removed. After the microtomographic analyses, a stability test with periotest and removal torque values were evaluated.

### Micro-computed tomography (CT) analysis

In this study, a micro-CT (SkyScan-1272, Bruker, Kontich, Belgium) device at Erciyes University Faculty of Dentistry Research Laboratories was used. Features of micro-CT device was used in this study; X-ray source; 20–100 kV, 10 W (< 5 μm spot size at 4W), X-ray camera; 16 MP, 4904 × 3280 px or 11 MP, 4032 × 2668, pixel size at maximum magnification; the expression < 0.35 μm for 16 MP camera is < 0.45 μm for 11 MP camera. The dental implant and bone contact surface area was examined in 2 to 3 dimensions, and the 2-mm area of the bone around the implant was measured. Before the implants were scanned, the micro-CT was set up to a 0.5-mm aluminum (Al)-copper (Cu) filter, 0.2° rotation state, 5-micron pixel size, 4 K resolution, and 360° shot. All implants were scanned at the same time. The NRecon v.1.6.3 software (Bruker-microBT, Belgium) program was used to transform the data obtained from the scanned implants, and the CTAn v.1.12 software (Bruker-micro-CT, Belgium) program was used for analysis. Images were reconstructed with NRecon 1.6.3 software (Bruker-micro-CT, Belgium) using 35 section hardening correction, 8 ring artifact correction, and minimum and maximum contrast limits, resulting in an average of 1800 cross-sections for each sample. All datasets were segmented and standardized with a user-defined global threshold. A fixed threshold range of 115 to 255 was applied to the image of all implants, and a threshold range of 35 to 255 was applied to the image of all bone tissue (CTAn v.1.12 software (Bruker-micro-CT, Belgium).

In micro-CT, percent intersection surface (IS/TS%; bone-implant contact surface), percent bone volume (BV/TV%; bone surface/total volume%), bone surface density (BS/TV mm^−1^; bone surface/total volume), connectivity density (Conn.Dn μm^−3^), total porosity percent (Po(tot)%), and trabecular thickness (Tb.Th μm) were measured. These data provided information on implant osseointegration and peri-implant trabecular microstructure.

### Periotest analysis

The Periotest M (Medizintechnik Gulden, Germany) device was used to assess the stability of the implants. Periotest measurement values between − 8 and + 50 are obtained. The lower the value indicated in the test, the better the stability of the measured tooth or implant [12]. The periotest device evaluates stabilization through a healing cap. Thus, to standardize the periostest measurements, all healing caps were set at 10 Ncm of torque with a rotary instrument. In this study, measurements were made at four different points: medial, distal, proximal, and lateral sides of the implants. Measurements were made twice, when implants were placed (T0) and after euthanization (T1); the obtained results were recorded.

### Removal torque test

The tibial samples were wrapped in saline solution-soaked gauze and stored at – 20 °C; they were then thawed at room temperature on the day of mechanical testing. Gradually increasing unscrewing torque was applied to the implants, and the peak torque value required for loosening the implants was recorded with a digital torque gauge (MARK-10 MTT01-12, NY, USA).

### Statistical analysis

The data of the study was reviewed by an independent statistician. Statistical analysis was performed with SPSS software (SPSS version 15, Chicago, IL, USA). Data regarding periotest, removal torque test, and micro-CT analysis were statistically analyzed with the Kruskal-Wallis non-parametric test, and multiple comparisons were made with the Mann-Whitney *U* test and one-way analysis of variance (ANOVA). Paired data from initial and final periotest measurements (T0 and T1) were analyzed with the Wilcoxon signed rank test and paired t test. A *p* value of .05 was considered significant.

## Results

### Removal torque testing tesults

The mean removal torque values of group shamOVX-LLLT (74.6 ± 15.4 Ncm) were significantly higher than the other groups (*p* < 0.05). In group OVX-LLLT, the mean removal torque value (56.1 ± 5.1 Ncm) were higher than that from group shamOVX (55.4 ± 18.5 Ncm). But there was no statistically significant difference (*p* = 0.92). The mean value of group OVX-LLLT (56.1 ± 5.1 Ncm) was significantly higher than that of group OVX (34.4 ± 7.4) (*p* < 0.05) (Table 1).

**Table 1:** The mean of the RTQ (removal torque) values and the difference between the groups

Removal torque results RTQ (Ncm)	*The mean of the RTQ (removal torque) values. Standard deviation (SD) and number of subjects (n)*					
	***Group shamOVX-LLLT*** ***[mean ± SD (n)]***		***Group shamOVX*** ***[mean ± SD (n)]***	***Group OVX-LLLT*** ***[mean ± SD (n)]***		***Group OVX*** ***[Mean ± SD (*n*)]***
	74.6 ± 15.4 (6)		55.4 ± 18.5 (6)	56.1 ± 5.1 (6)		34.4 ± 7.4 (6)
	***Differences in mean values of RTQ values between groups***					
	***Group*** ***ShamOVX(LLLT)-OVX***	***Group*** ***ShamOVX(LLLT)-ShamOVX***	***Group*** ***ShamOVX(LLLT) -OVX(LLLT)***	***Group*** ***OVX(LLLT)-OVX***	***Group*** ***ShamOVX–OVX(LLLT)***	***Group*** ***ShamOVX-OVX***
	40.1**	19.2*	18.5*	21.6*	0.71	20.9*

*Statistical difference between groups, *p* < 0.05

**Statistical difference between groups, *p* < 0.001

### Periotest stability results

The mean T0 (implantation day) value for group shamOVX-LLLT, group shamOVX, groupOVX-LLLT, and groupOVX were as follows: − 5.60 (shamOVX-LLLT), − 5.33 (shamOVX), − 1.39 (groupOVX-LLLT), and − 1.07 (groupOVX). At T1(euthanized day) values, the mean values obtained were as follows: − 7.22 (group sham OVX-LLLT), − 5.80 (group shamOVX), − 6.25 (group OVX-LLLT), and − 3.88 (group OVX).

The mean T1 value was found to be significantly higher than T0 in group shamOVX-LLLT (*p* = 0.01). In group shamOVX, the T1 mean was higher than that at T0, but this difference was not statistically significant (*p* = 0.44). The mean T1 value was significantly higher than that at T0 in group OVX-LLT (*p* < 0.001). The mean T1 values of group OVX were significantly higher than at T0 (*p* < 0.001). The greatest difference between T1 and T0 was found in the osteoporotic laser group (group OVX-LLLT) (Table 2).

**Table 2:** The mean periotest values at T0 (implantation day) and T1 (euthanization day) periods, intra-group comparison of T1-T0 differences and comparison of T0 and T1 values between groups

*The mean of periotest values at T0 (implantation day) and T1 (euthanization day) periods. Standard deviation (SD) and number of subjects (n)*						
	***Group shamOVX-LLLT*** ***[mean*** ± SD ***(n)]***	***Group shamOVX*** ***[mean*** ± SD ***(n)]***		***Group OVX-LLLT*** ***[mean*** ± SD ***(n)]***	***Group (OVX)*** ***[mean*** ± SD ***(n)]***	
Periotest T0	− 5.60 ± 1.6(11)	− 5.33 ± 2.1(11)		− 1.39 ± 1.2(11)	− 1.07 ± 0.6(11)	
Periotest T1	− 7.2 ± 0.6(11)	− 5.8 ± 0.5(11)		− 6.2 ± 0.5(11)	− 3.8 ± 0.9(11)	
*Comparison of the differences of T1-T0 periotest values*						
Periotest T1-T0	***Group shamOVX-LLLT*** **(T1-T0)**	***Group shamOVX*** **(T1-T0)**		***Group OVX-LLLT*** **(T1-T0)**	***Group (OVX)*** **(T1-T0)**	
	− 1.6*	− 0.47		− 5.2**	− 2.8**	
*The differences and comparison of the T0 and T1 values between groups*						
	***Group*** ***ShamOVX(LLLT)-OVX***	***Group*** ***ShamOVX(LLLT)-ShamOVX***	***Group*** ***ShamOVX(LLLT)-OVX(LLLT)***	***Group*** ***OVX(LLLT)-OVX***	***Group*** ***ShamOVX-OVX(LLLT)***	***Group*** ***ShamOVX-OVX***
Periotest T0	− 4.5**	− 0.2	− 4.2**	− 0.3	− 3.9**	− 4.2**
Periotest T1	− 3.3**	− 1.4**	− 0.9*	− 2.3**	− 0.4	− 1.9**

Standard deviation (SD), number of subjects (*n*)

*Statistical difference between groups, *p* < 0.05

**Statistical difference between groups, *p* < 0.001

The mean values of T0 between the shamOVX-LLLT and shamOVX groups were not significant (*p* = 0.67). Also, the mean values of T0 between the OVX-LLLT and OVX groups were not significant (*p* = 0.62). The mean values of T0 between the ovariectomized and non-ovariectomized groups were statistically significant (*p* < 0.05). The mean T1 value of group shamOVX-LLLT was significantly higher than the mean T1 of all other groups (*p* < 0.05). The difference between the T1 periotest values of group shamOVX and group OVX-LLLT was not statistically significant (*p* = 0.14) (Table 2).

### Micro-CT result

**Fig. 2:**
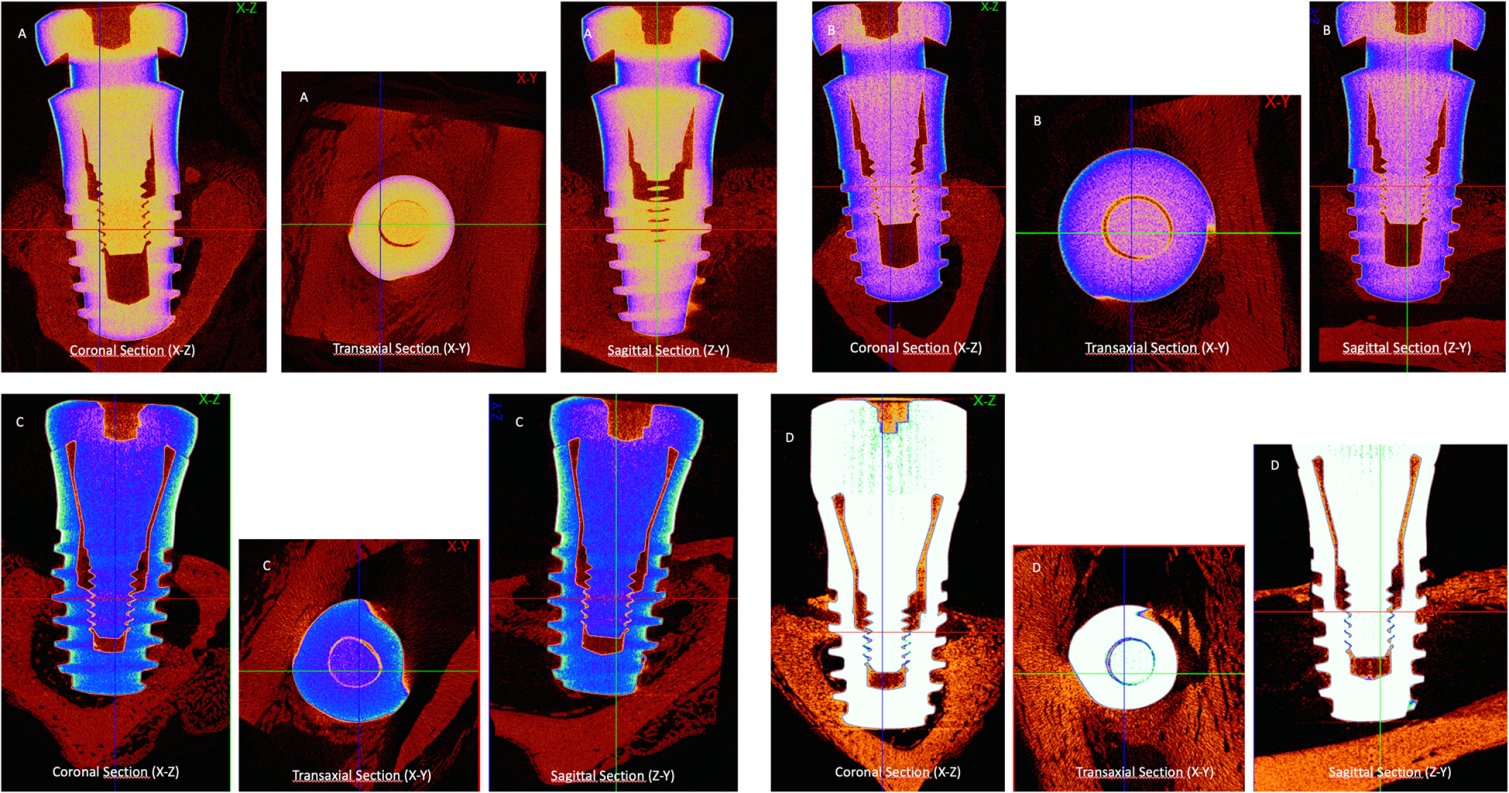
Images of coronal (X-Z), transaxial (X-Y), and sagittal (Z-Y) sections of the groups in the micro-CT scans. Note the bone implant contact differences among the groups. **a** ShamOVX-LLLT, **b** ShamOVX, **c** OVX-LLLT, and **d** OVX

**Fig. 3:**
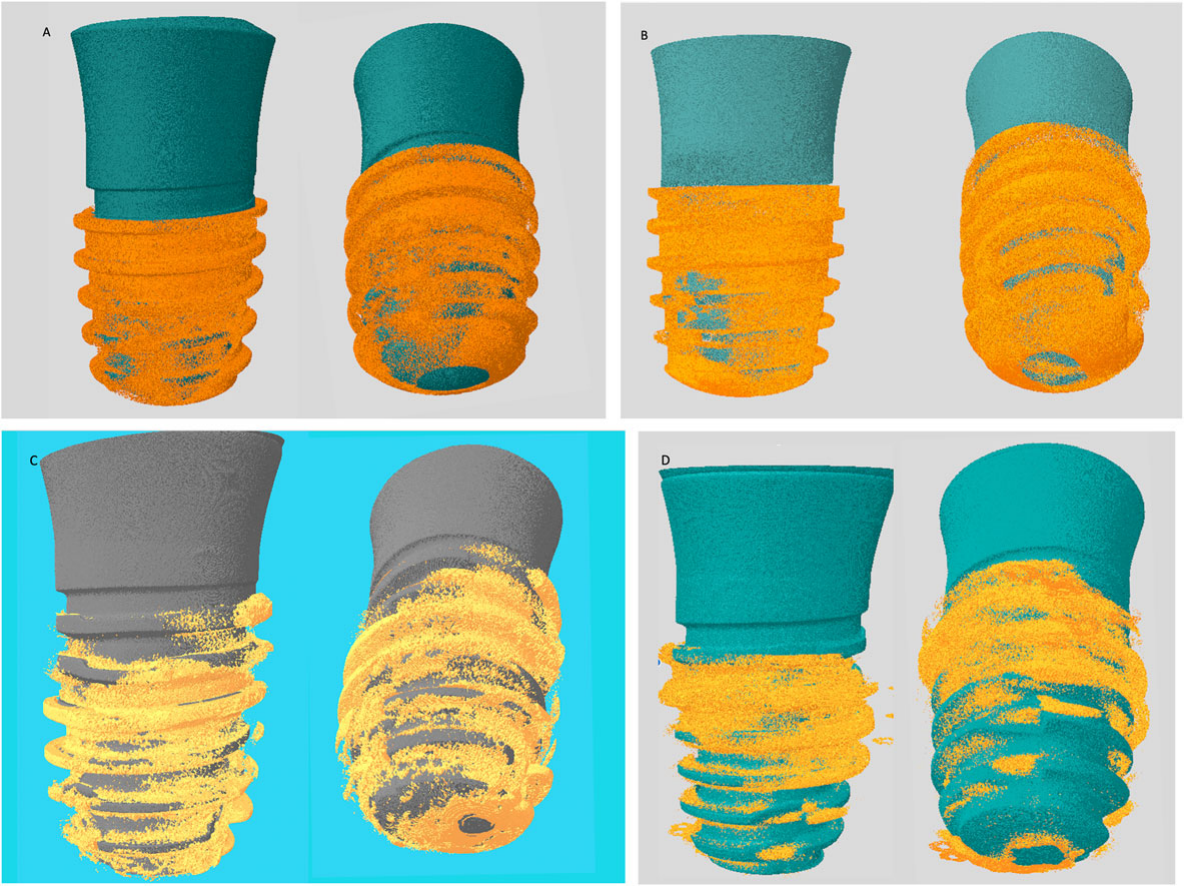
3D images of the new bone formation at peri-implant area in the groups obtained from micro-CT analysis. Note that the increase in the bone volume of the osteoporotic bone around the implant treated with LLLT in OVX-LLLT compared with OVX. **a** ShamOVX-LLLT, **b** ShamOVX, **c** OVX-LLLT, **d** OVX

The high-resolution 3D images and 2D graphs obtained from micro-CT clearly depicted the differences among the four groups, and the data are shown in Table 3. These results also show the information on implant osseointegration and peri-implant trabecular microstructure (Fig. 2 and Fig. 3).

**Table 3:** The mean of micro-CT parameters according to groups

	Group shamOVX-LLLT [mean ± SD (*n*)]	Group shamOVX [mean ± SD (*n*)]	Group OVX-LLLT [mean ± SD (*n*)]	Group OVX [mean ± SD (*n*)]
Percent intersection surface (IS/TS%)	51.1 ± 3.2 (5)	45.6 ± 3.9 (5)	43.5 ± 3.1 (5)	35.3 ± 6.1 (5)
Percent of bone volume (BV/TV%)	47.7 ± 2.2 (5)	41.8 ± 6.1 (5)	35.7 ± 6.02 (5)	28.3 ± 8.9 (5)
Bone surface density (BS/TV mm^−1^)	42.8 ± 3.8 (5)	37.7 ± 9.4 (5)	31.8 ± 10.08 (5)	26.5 ± 8.2 (5)
Connectivity density (Conn.Dn μm^−3^)	7.6 ± 1.8 (5)	6.5 ± 2.8 (5)	4.7 ± 2.2 (5)	3.5 ± 2.9 (5)
Total porosity percent (Po(tot)%)	52.2 ± 2.2 (5)	58.1 ± 6.1 (5)	64.2 ± 6.02 (5)	71.6 ± 8.9 (5)
Trabecular thickness (Tb.Th μm)	156.3 ± 20.5 (5)	124.1 ± 20.03 (5)	82.06 ± 23.7 (5)	69.2 ± 30.2 (5)

Mean, standard deviation (*SD*) and number of subjects (*n*)

### Percent intersection surface (bone-implant contact surface) (IS/TS%)

The results of the analysis showed that the values in group OVX were lower than those in group shamOVX. The cause of this result was thought to be a decrease in bone quality and density due to the formation of osteoporosis. The mean percent intersection surface values of group shamOVX-LLLT (51.1 ± 3.2%) were higher than those of all groups. The mean of group OVX-LLLT (43.5 ± 3.1%) was lower than the mean of group shamOVX-LLLT (51.1 ± 3.2%) and group shamOVX (45.6 ± 3.9%). However, the mean value of group OVX-LLLT was close to group shamOVX, and the difference between these two groups was not statistically significant (*p* = 0.4). In group OVX, the percent intersection surface means (35.3 ± 6.1%) were significantly lower than those in all other groups (*p* < 0.05) (Tables 3 and 4). This result was attributed to osteoporosis formation.

**Table 4:** Differences in mean values of micro-CT parameters between groups

	Group ShO(L)-OVX	Group ShO(L)-ShO	Group ShO(L)-OVX(L)	Group OVX(L)-OVX	Group ShO-OVX(L)	Group ShO-OVX
Percent intersection surface (IS/TS%)	15.7**	5.4	7.5*	8.2*	2.1	10.3*
Percent of bone volume (BV/TV%)	19.4**	5.9	12.07*	7.3	6.09	13.4*
Bone surface density (BS/TV mm^−1^)	16.2*	5.1	11.05*	5.2	5.9	11.1*
Connectivity density (Conn.Dn μm^−3^)	4*	1.02	2.8	1.2	1.8	3.04
Total porosity percent (Po(tot)%)	19.4**	5.9	12.08*	7.3	6.1	13.4*
Trabecular thickness (Tb.Th μm)	87.04**	32.1	74.2**	12.8	42.03*	54.8*

*Statistical difference between groups, *p* < 0.05

**Statistical difference between groups, *p* < 0.001

*ShO(L)* group shamOVX-LLLT, *ShO* group shamOVX, *OVX(L)* group OVX-LLLT, *OVX* group OVX

### Percentage of bone volume (BV/TV%)

The difference of percent bone volume between group shamOVX-LLLT and OVX (*p* < 0.001), group shamOVX-LLLT and OVX-LLLT (*p* = 0.009), and group shamOVX and OVX (*p* = 0.004) was statistically significant. The mean percent bone volume values of group shamOVX-LLLT (47.7 ± 2.2%) were higher than those of the other groups. It was determined that obtained values of group OVX (28.3 ± 8.93%) were lower than those of all the other groups, and the decrease in bone quality and density due to osteoporosis was considered to have resulted from this. The mean of group OVX-LLLT values (35.7 ± 6.02%) was lower than that of closed group shamOVX (41.8 ± 6.1%), but the difference was not statistically significant (p=0.14) (Tables 3 and 4).

### Bone surface density (BS/TV mm^−1^)

Bone surface density was examined, and the mean of group shamOVX-LLLT values (42.8 ± 3.8 mm^−1^) was higher than those of all the groups. The mean of group OVX values (26.5 ± 8.2 mm^−1^) was the lowest. These results also support osteoporosis. Statistical analysis between the groups showed there was a significant difference between groups shamOVX-LLLT and OVX, groups shamOVX-LLLT and OVX-LLLT, and groups shamOVX and OVX (*p* < 0.05). The differences between the other groups were not statistically significant (Tables 3 and 4).

### Connectivity density (Conn. Dn μm^−3^)

As a result of connectivity density analysis, the difference between groups shamOVX-LLLT and OVX was found to be significant (*p* = 0.03). The highest mean values of connectivity density were in group shamOVX-LLLT (7.6 ± 1.8 μm^−3^). The lowest value was obtained in group OVX (3.5 ± 2.9 μm^−3^) (Tables 3 and 4).

### Total porosity percent (Po (tot)%)

Total porosity percent analysis showed that the difference between groups shamOVX-LLLT and OVX, groups shamOVX-LLLT and OVX-LLLT, and groups shamOVX and OVX was statistically significant (*p* < 0.05). Also, the difference between the groups OVX-LLLT and the shamOVX was not significant (*p* = 0.14). The mean of the group OVX-LLLT total porosity percent (64.2 ± 6.02) was similar to the mean of group shamOVX (58.1 ± 6.1). The mean of the group shamOVX-LLLT total porosity percent values (52.2 ± 2.2%) was lower than all groups. The mean of the group OVX values (71.6 ± 8.9%) was higher than those of other groups. This result was parallel to the formation of the osteoporosis model (Tables 3 and 4).

### Trabecular thickness (Tb.Th μm)

Trabecular thickness analysis showed that the mean trabecular thickness value (156.3 ± 20.5 μm) of group shamOVX-LLLT was higher than that in the other groups. The mean of group OVX (69.2 ± 30.2 μm) was the lowest of all groups. According to these results, the trabecular thickness in the group of osteoporosis decreased significantly (*p* < 0.001). Although the mean trabecular thickness values of group OVX-LLLT (82.06 ± 23.7 μm) was higher than group OVX (69.2 *±* 30.2 μm), the difference was not statistically significant (*p* = 0.4). The difference between the other groups was statistically significant (*p* < 0.05). Trabecular thickness analysis was showed that the mean of group shamOVX-LLLT (24.5 ± 9.8 μm) was lower than all other groups (Tables 3 and 4).

## Discussion

Several methods have been developed to increase dental implant osseointegrations. These include mechanical methods, such as modifications of implant surfaces or coating with medical agents, and the methods used to increase biostimulation include vibration and LLLT applications [13–16]. Among these methods, LLLT is one of the more promising methods to improve osseointegration of biomaterials and to prepare an appropriate implant area [8]. Liu et al. indicated that LLLT accelerated fracture healing and increased callus volume, especially at early stages of bone healing [17]. In in vivo comparative studies involving cell cultures, LLLT was found to have positive effects on biostimulation of cells [18]. It has been reported that LLLT may accelerate resorption or formation activities depending on the stages of bone fracture repair [19]. Given the evidence of positive effects of LLLT on bone healing, it is possible to accelerate or increase the osseointegration of implants with LLLT. Khandra et al. pointed out that the osseointegrations of titanium implants accelerated in their studies with LLLT [13]. In the literature, there are a few studies in which dental implants and LLLT are applied in osteoporotic bones. In this study, the authors emphasized that LLLT positively contributed to osseointegration of implants applied to osteopenic rats [20].

It has recently been reported that LLLT should not exceed 1 W of output power for biostimulation [21]. Biostimulation has been shown to increase in studies with 0.3 W output power [22–24]. According to this information, 0.3 W of output power is applied in our study. For LLLT, it is stated that the ideal wavelength is from 550 to 950 nm [25]. We used 940 nm at the wavelength diode laser in our study. It was not possible to determine an effective dose on bone tissue, and very different doses were used in the literature [21]. The LLLT protocol we applied in our study is similar to that of Khadara et al. [13]. Factors determining the effect of the laser on the tissues include the tissue that is administered and the penetrating dose. The laser energy transmitted per cm^2^ area in the target tissue is called energy or dose [21]. The dose was standardized according to the tissues, but since the effective wavelength on the bone tissue of the LLLT was not standard, no protocol could be established [26]. It was seen that an effective dose could not be determined on bone tissue, but different doses were used in the literature. Similar studies in the literature show that 3 to 10 J/cm^2^ energy is used for bone tissue [13, 19, 27]. In this study, 6 J/cm^2^ energy was used.

By using some digital test devices such as periotest, resonance frequency analysis can be used to evaluate implant stability [28]. There are many studies in the literature that are used in both devices [29–36]. When the literature is examined, periotest is stated to be a reliable device in the diagnosis of implant stability, and current studies using the periotest device are available [32–36]. Also, periotest is a non-invasive diagnostic method that evaluates the stability between the implant surface and the bone [37]. It also has the advantages of being easily portable, ergonomic, cost-effective, practical, and self-sufficient in measuring [12]. Besides these advantages, the fact that it has been used in many studies in the literature has been a factor in the preference of the periotest device in this study. The measurements have been repeated twice. A literature review shows that implants applied to osteoporosis-generated models give lower results in stability tests [38–41]. In the present study, in the group with osteoporosis, the stability test results obtained were lower. Following LLLT, the value in the osteoporosis group exceeds the value of a healthy and non-laser-treated group. The highest increase was obtained in group OVX-LLLT.

Recent studies showed that the mean of the removal torque values of the implants applied to the osteoporosis model bones is lower than the mean of the implants applied to healthy groups [38–41]. The results of this study are consistent with the literature. The mean of the removal torque values of the implants in the sham-ovariectomy groups was higher in group shamOVX-LLLT than group shamOVX. In the osteoporosis group, the mean values of the removal torque were higher in group OVX-LLLT than the group OVX. The mean values of group OVX-LLLT (56.1 ± 5.1) and shamOVX (55.4 ± 18.5) were similar, and the difference between them was not statistically significant (*p* = 0.92). Also, the mean of group shamOVX-LLLT was significantly higher than that of all other groups. The removal torque results showed that LLLT application contributed to osseointegration in both osteoporosis and healthy bones. There is a strong, positive, and statistically significant correlation between the percentage of bone volume and RTQ variables (*r* 0.62 and *p* 0.003). This result showed that the amount of bone around the implant and the increase of RTQ values were parallel to each other. These values were the lowest in rabbits with experimentally induced osteoporosis.

In determining bone quality, bone density alone does not provide clear information. To predict bone strength and fracture resistance, bone should be considered together with micro-structure [42]. Currently, histomorphometric evaluation and micro-CT examination are included in the evaluation of the quality and density of the bone. As stated in the literature, histomorphometry is still known today as the most effective method for determining bone-implant contact (BIC) and bone formation at a cell level. Also, BIC and peri-implant bone density (BV/TV) were traditionally obtained by histology and serve biological implant integration [43].. Nevertheless, histomorphometry is a destructive method, and the same sample cannot be used in the evaluation of other tests such as removal torque and stability evaluation [44]. In particular, the data obtained in evaluating new bone formation are limited to 2D images data do not reflect the actual 3D structure of bone [44]. In addition, the process of completion of the analysis is quite long [43]. Therefore, a non-invasive method is needed to evaluate osseointegration. Microcomputer tomography (micro-CT) has taken its place in the literature as a non-destructive technique that allows precise quantitative three-dimensional (3D) analysis. Micro-CT is accepted as the gold standard for assessment of trabecular microstructure [45]. The pixel, which forms the 2D or 3D cross-sectional images obtained by micro-CT, is in the micro dimension, allowing the internal structure of a material to be displayed in three dimensions in a non-destructive manner and the measurements made accordingly [46]. Micro-CT can analyze the data for the bone up to a few microns on the implant surface and in the periimplant area and evaluate both the qualitative and quantitative morphometry of implant bone integration [47]. Despite all advantages, there are studies indicating that micro-CT is not as effective as histomorphometry in evaluating bone-implant contact. Thus, in this study, both biomechanical tests and morphometric analyses and bone-implant contact obtained from micro-CT were used to evaluate osseointegration. However, there are also micro-CT studies in which the connection in the bone implant interface is evaluated by BIC, BV/TV, trabecular thickness, trabecular number, and trabecular separation [48–50].

There are studies in which micro-CT and histomorphometric analysis have been used to evaluate dental implant osseointegration comparatively [43, 51, 52]. He, Tao et al. In their current study, compared bone density using micro-CT and Histomorphometric analysis and stated that there was a correlation among them [43]. In the studies of Vandeweghe et al., they compared the bone density and bone-implant contact of micro-CT and Histomorphometry, it was found that there was a correlation between the data [51]. Diefenbeck et al.’s study, it was evaluated osseointegration, they reported that bone-implant contact (BIC) showed good correlation with biomechanical tests and was a more important parameter than bone area density (BA) in evaluating osseointegration [53]. Also, in Bissinger et al. they compared the bone-implant contact (BIC%) of micro-CT and histomorphometry, it was found that there was a correlation between the data too [52]. In the present study, a similar correlation was obtained between BIC value and biomechanical tests. It was seen that the obtained results are compatible with the literature. There is a strong, positive, and statistically significant correlation between the percentage of bone volume (BV/TV%) and RTQ variables (*r* 0.62 and *p* 0.003) and between the percentage of bone volume (BV/TV%) and bone-implant contact (BIC%) (*r* 0.75 and *p* < 0.001), and there is a positive, moderate, and statistically significant correlation between the bone-implant contact (BIC%) and RTQ variables (*r* 0.59 and *p* 0.006) (Table 5).

**Table 5:** Correlation analysis

	Correlation	***p*** value
**BV/TV% (percentage of bone volume): RTQ Ncm (removal torque analysis)**	0.62	0.003
**BV/TV% (percentage of bone volume): IS/TS% (bone-implant contact surface)**	0.75	*p* < 0.001
**IS/TS% (bone-implant contact surface): RTQ Ncm (removal torque analysis)**	0.59	0.006

In similar studies with micro-CT, percent intersection surface, percent bone volume, bone surface density, connectivity density, trabecular thickness, and trabecular separation parameters were investigated [54–59]. These values have also been examined in the present study. In this study, the percent bone volume value parallel to other studies. Percent bone volume values in the group OVX-LLLT, which was osteoporotic bone treated with LLLT, was higher than in the osteoporotic control group (group OVX) and close to the healthy control group (group shamOVX). These results showed that there is a positive effect of LLLT on osteoporotic bones.

In this study, bone-implant contact values were higher in the healthy bone treated with LLLT (group shamOVX-LLLT) than in the healthy control group (group shamOVX), in osteoporotic bone treated with LLLT (group OVX-LLLT), and in the osteoporotic control group (group OVX). The difference between the osteoporotic bone treated with LLLT and the osteoporotic control groups was found to be statistically significant, and the mean values of the group OVX-LLLT came close to those of the healthy control group. These results support a positive effect of LLLT on osseointegration of implant in osteoporotic bones.

Trabecular thickness, bone surface density, total porosity percent, and total volume of pore space values are used as the markers of osteoporosis in micro-CT analyses. Some studies showed that trabecular thickness and bone surface density values decreased, whereas total porosity percent and the total volume of pore space values increased in osteoporotic bones [55, 57]. In this study, the trabecular thickness and bone surface density values decreased, whereas the total porosity percentage and total volume of pore space values increased in the ovariectomized rabbits compared with the healthy group. These values in the osteoporosis group treated with LLLT were close to the values of the healthy control group in micro-CT analysis.

## Conclusions and declarations

In this experimental study, all values obtained from biomechanical tests and micro-CT in the OVX-LLLT group were significantly higher than that of the OVX group and achieved the values in the healthy bone. These results show that LLLT has a positive effect on osseointegration of implants placed in osteoporotic bone. The fact that laser therapy is a non-invasive technique, there are studies with positive effects, this study also supports positive effects, and may provide more clinical studies on the effects of low-dose laser therapy on implant osseointegration. Further clinical trials are needed to evaluate the clinical efficacy and safety of the results obtained from this experimental study.


**Additional file 1.** Video images obtained from Micro-CT.

## Abbreviations

LLLT: Low-level laser therapy
ShamOVX: Sham ovariectomy
OVX: Ovariectomy
IS/TS: Bone-implant contact surface
BV/TV: Percent of bone volume
BS/TS: Bone surface density
Conn. Dn: Connectivity density
Po (tot): Total porosity percent
Tb.Th: Trabecular thickness
